# 1405. Angel of Death - Tetanus and Rabies - Indian Experience

**DOI:** 10.1093/ofid/ofad500.1242

**Published:** 2023-11-27

**Authors:** V S Srikanth

**Affiliations:** 15AFH, jaisalmer, Rajasthan, India

## Abstract

**Background:**

Tetanus is an old-world disease and Rabies dated back to the Vedic period i.e 1500-500BC in ancient Indian scripture, Yama, the mythical God of Death. This is one of very few recent studies done in India on Tetanus and Rabies, the data available on Tetanus and rabies are less so we are trying to share our experience on these diseases, so it will help the physicians to get a better understanding.

**Methods:**

This is a retrospective study, detailed 3 years case sheet review was done and the patient’s clinical presentation and prognosis was noted in pre designed format.Inclusion criteria – All diagnosed cases of tetanus and rabies,Exclusion criteria – patient already received treatment from local hospital.

**Results:**

58 tetanus cases with average duration hospital stay 15 days, common occupation farmers(barefoot workers).The site of injury -foot in 65% cases followed by injuries to the fingers or the hand in 30% and 5%cases due to injury while tooth picking with pin, splinter removal using pins.Clinical symptoms -Trismus“Lock jaw”(41),difficulty in walking (2),limb pain/stiffness(17),back muscle, pain/stiffness(12,)Dysphagia (7) ,72% autonomic dysfunction.Opisthotonos position and riscus sardonicus developed after 7-8days of infection. 18% mortality was noted most cases were unvaccinated cases. 31 Rabies cases - age group of 35-45, most cases were unprovoked bite.Common site of injury lower limbs.68% grade 3, 32% grade.Symptoms from bite1 months High grade fever, anxiety were initial symptoms followed by hydrophobia and aerophobia. 20% cases had seizure. Minimum hospital stays 12 days,Mortality 100.

**Conclusion:**

Tetanus and rabies are preventable diseases; if ignored can cost life .So Rabies vaccination and immunoglobulin should be administered on time

**Disclosures:**

**All Authors**: No reported disclosures

